# A guide to inter-joint coordination characterization for discrete movements: a comparative study

**DOI:** 10.1186/s12984-023-01252-2

**Published:** 2023-09-30

**Authors:** Océane Dubois, Agnès Roby-Brami, Ross Parry, Mahdi Khoramshahi, Nathanaël Jarrassé

**Affiliations:** 1 Institute of Intelligent Systems and Robotics (CNRS-UMR 7222), University Pierre & Marie Curie, Paris, France; 2 LINP2, UPL, UFR STAPS, University Paris Nanterre, 200 Avenue de la République, 92001 Nanterre, France

**Keywords:** Inter-joint coordination, Discrete tasks, Kinematic measurement, Multiple joints, Upper-limb coordination

## Abstract

Characterizing human movement is essential for understanding movement disorders, evaluating progress in rehabilitation, or even analyzing how a person adapts to the use of assistive devices. Thanks to the improvement of motion capture technology, recording human movement has become increasingly accessible and easier to conduct. Over the last few years, multiple methods have been proposed for characterizing inter-joint coordination. Despite this, there is no real consensus regarding how these different inter-joint coordination metrics should be applied when analyzing the coordination of discrete movement from kinematic data. In this work, we consider 12 coordination metrics identified from the literature and apply them to a simulated dataset based on reaching movements using two degrees of freedom. Each metric is evaluated according to eight criteria based on current understanding of human motor control physiology, i.e, each metric is graded on how well it fulfills each of these criteria. This comparative analysis highlights that no single inter-joint coordination metric can be considered as ideal. Depending on the movement characteristics that one seeks to understand, one or several metrics among those reviewed here may be pertinent in data analysis. We propose four main factors when choosing a metric (or a group of metrics): the importance of temporal vs. spatial coordination, the need for result explainability, the size of the dataset, and the computational resources. As a result, this study shows that extracting the relevant characteristics of inter-joint coordination is a scientific challenge and requires a methodical choice. As this preliminary study is conducted on a limited dataset, a more comprehensive analysis, introducing more variability, could be complementary to these results.

## Introduction

As a result of technological improvements over the last years (smaller, cheaper, more precise sensors), motion capture has become an accessible and data-rich technique. The kinematic analysis of human body movement is extensively used in numerous fundamental studies on the physiology of human motricity and various applied fields, in particular rehabilitation. However, most previous studies were devoted to lower limbs and gait, while upper-limb motion analysis was relatively overlooked. Furthermore, most studies in upper-limb motion analysis are limited to end-point displacement (i.e., the hand or the tip of a finger) in space or qualitative descriptions. The lack of a relevant quantitative metric to analyze upper-limb movements hinders tracking the evolution of human subjects during learning, rehabilitation, or development. Therefore, the present study investigates the capacity of several metrics in evaluating inter-joint coordination through a systematic review and experimental study. The presentation and discussion of those metrics are first based on the fundamental knowledge of human motor control.

### Origins of motion capture

The pioneering works by Marey [1] who invented chronophotography, allowed the temporal sampling of body posture snapshots and led to the precise description of human and animal bodily movements. These works were further developed by NA Bernstein who improved the recording techniques, to sample the movement in time as a succession of different points position linked to anatomical markers. He theorized the physiology of motor control in the early 30 s’, lately translated into English [2]. According to Bernstein [3], movement organization is hierarchical. He distinguishes four levels: the task-related action level, the goal-oriented spatial level, the organization of joint and muscle activity, and the tonus. Although this primitive description has been strongly questioned afterward, it presents a practical framework for understanding movement organization. Within the present article, we shall concentrate on the questions of inter-joint coordination to generate a spatially oriented movement of the upper limb.

### End-effector trajectory in space

Nowadays, motion capture systems allow the collection of big amounts of data on segments and joints during a movement using optical or non-optical (inertial, magnetic or stretch sensors) systems. Fundamental progress in human motor control is supported by theoretical approaches to computational control and robotic modelling. Experimental studies on fast upper-limb goal-directed movements lead to the agreement that they are characterized by a roughly straight trajectory of the end-point of the kinematic chain (i.e, the tip of the finger or the end-effector of a manipulandum in a pointing task) with a smooth velocity profile [4]. This pattern, primarily interpreted as an optimization [5], is now attributed to stochastic optimal control [6], (see also [7, 8] for review). Whatever the fundamental open questions, the endpoint kinematic variables (such as maximum speed, completion time, smoothness, length, and curvature of the trajectory) are widely used as metrics to characterize human movement in many applicative fields. Indeed, end-point metrics are easy to record, compute, and interpret since they reduce the movement to one point moving in space and time. For example, they are commonly used to monitor improvements in upper-limb motor function through the course of rehabilitation [9–12], the child development (e.g. [13]) or the level of skill in sports (e.g. [14]).

### Joint rotation contribution

However, due to the large redundancy of the human arm, different patterns of upper-limb joint contribution can be used to perform the same end-effector trajectory [2]. Redundancy, means that more degrees of freedom are available than necessary to achieve a task. Increasing the number of degrees of freedom for a task also increases the variability of possible coordination. This question is known as the ill-posed inverse kinematics problem in robotics and has prompted many experimental and theoretical work. Early experimental studies showed that the pattern of joint contribution is individual and quite reproducible for each target in space [15] but failed to find biomechanical correlates of joint contribution (e.g. [16, 17]). Optimal control modelling states that a unique solution to an ill-posed problem can be obtained corresponding to a minimum of a cost function. However, the redundancy problem has rarely been modelled and only with additional restrictive conditions [6, 18].

### Interjoint coordination

It is currently and subjectively admitted that “good” inter-joint coordination is essential to upper-limb function. However, the multidisciplinary concept of inter-joint coordination needs clarification. As underlined by Shirota et al., inter-joint coordination remains a common term used in rehabilitation, engineering, and neuroscience but “common agreement on what should be measured” is generally lacking [19]. Bernstein’s first definition describes inter-joint coordination as “mastering redundant degrees of freedom of the moving organ” [2]. For Bernstein, inter-joint coordination is the ability to choose one possibility among all possible solutions to accomplish the task. Since the 2000 s and the development of precise sensors to measure joint position in space and time, several other definitions of inter-joint coordination have emerged. An analogous definition to Bernstein’s was elaborated by [20] as “the ability to produce complex movements involving several limbs and/or joints.”

Other authors motivated by the mathematics of dynamical complex systems focused on temporal coordination (review in [21]). The idea of a spatiotemporal inter-joint organization is expressed as “coordination is not just matter in motion, rather, coordination is a functional spatio-temporal order” [22]. A more specific definition was presented for locomotor coordination by defining coordination as “an ability to maintain a context-dependent and phase-dependent cyclical relationship between different body segments or joints in both spatial and temporal domains” [23]. This last definition was then extended to all types of movements, and the idea of a goal-specific coordination was added as “A spatio-temporal relationship between kinematic, kinetic and physiological variables of two or more limbs executing a motor task with a common goal” [19]. A similar definition has been suggested by [24] as “A goal-oriented process in which joint degrees of freedom are organized in spatial and temporal domains such that the end-point reaches a desired location in a context-dependent manner”. The “location” designates both the position and the orientation of the end-point.

The concept of synergy is closely related to that of coordination and has also been used to describe different phenomena. According to [2] synergies are fundamental building blocks of motor control, which combine several elements, sharing the same spatio-temporal properties and “work together” [25], further underlined that synergies are not “hardwired” but task related combinations of elements. They are endowed with properties of flexibility and automatic compensation between elements in order to stabilize the important task-related variables. Those properties are formalized by the Uncontrolled Manifold (UCM) model [26]. The UCM algorithm decomposes the joint velocities into the controlled task-space and the uncontrolled null-space. Null-space velocities (or self motion) do not create end-effector motions but afford automatic compensation between joints.

### Measuring versus evaluating

To date, inter-joint coordination metrics are much less used than end-effector metrics. Measuring human motor control during task performance generally infers, classifying movements subjectively as “good” or “bad”. In this classification process, a “standard movement” is taken to be a “good movement” with relative success determined as the extent to which an individual is able to reproduce this configuration. In the absence of a clear definition, standard movement is usually defined by the behavior exhibited by a group of people considered “healthy” [27], this is the “normal” condition. Accordingly, movements which are found to deviate significantly from this reference movement might be considered as pathological. This way of proceeding has been repudiated by [28], who argued that a movement that differs from the majority should not be necessarily considered as pathological by reference to “normal” but rather as “atypical”. To them, this means that the Central Nervous System (CNS) has selected a solution that minimizes the costs of the movement (i.e, the time cost, the energy cost, or the cost generated by impaired muscles). As a consequence, the selected movement is the optimal one for this person, at this moment.

In a clinical setting, inter-joint coordination should be carefully analyzed. For example, inter-joint coordination is drastically altered in stroke patients. They show a disruption of the usual, flexible shoulder-elbow coordination [29], tending to exhibit stereotypical patterns of joint coupling, often referred to as pathological fixed synergies [30–32]. In addition, they may exhibit new motor strategies with trunk flexion in order to compensate the impairment of shoulder flexion and elbow extension ([33] review in [34]). Such compensatory movements are immediately efficient since they allow the patient to complete the task (e.g. grasp objects and perform daily life activities). However, they are considered as “bad coordination” by physicians and physiotherapists on a long-term perspective since they may induce learned nonuse phenomenon [35, 36]. Trunk flexion to compensate elbow extension impairment is relatively easy to interpret as a function of workspace amplitude but this is not the case when compensatory strategies are more complicated, for example during daily life activity, or when the impairment is more complex. More generally, compensatory movements may contribute to orthopedic or musculoskeletal disorders, for example in amputees using a transradial prosthesis [37, 38]. In these cases, physiotherapists need to work with the patient, to help them to learn or relearn patterns of joint rotations to decrease the predictable constraints. There is a crucial need of metrics accepted by all in order to distinguish those features directly linked to the impairment and those which are due to compensatory behavior, in order to properly manage and monitor rehabilitation exercises and to follow-up recovery. Other applicative fields are also keen to use inter-joint metrics, for example to quantify the acquisition of skilled gestures in sport [39–41], or to evaluate a robotic ergonomic assistance [42].

### Delimitation of the case of study

The selected metrics that are presented here can be applied to upper-arm kinematic data of discrete movements. Kinematic data describe a given movement (position, velocity, acceleration) without considering the forces generating them [43]. They can be acquired via an optical motion capture system (camera, infrared camera, and markers) or accelerometers, electrogoniometers, etc. These different data acquisition methods have already been widely used to quantify the severity of movement disorders [44]. These data are easy to record, and measurement systems are non-invasive. Metrics based on EMG or force signals are excluded from the present review.

Discrete movements are “bounded by distinct postures” [45]. For example, reaching an object, reaching out to open a door, or shaking a hand are examples of discrete movements. These movements are opposed to cyclical or rhythmical movements (such as gait) that are generally more conducive to specific metrics that evaluate recurrent aspects of movement, such as wavelet decomposition [46], analysis of the power spectrum of the signal [47], Fourier phase [48] etc. that will not be considered here.

## Method

A literature review has been conducted in order to extract the main inter-joint coordination metrics based on kinematic data for discrete movements. Subsequently, these metrics were applied to an experimental dataset to assess their efficacy in distinguishing various coordination strategies.

### Review methodology

Scientific studies were selected from the PubMed, ScienceDirect, and Google Scholar databases using the following keywords: *inter-joint coordination, upper-limb coordination, spatial* and *temporal coordination, coordination metrics, joint angle organization*. Interlimb coordination has not been investigated here.

An initial selection was made from the results, while a manual search strategy using references from the retained articles provided additional studies for analysis. Because this paper focuses on metrics for inter-joint coordination based on kinematic data, all papers which were exclusively based upon end-effector data, or other forms of biomechanical signals (EMGs, torque...) were excluded. Papers measuring coordination of cyclical tasks with metrics using specific characteristics of those tasks (such as frequency) were also removed. Some metrics, in the litterature, were used as well for upper-limb as for lower-limb, as long as they could characterize discrete kinematic movements, they were kept in the analysis.

In total, 67 studies were retained, all published before June 2022. A total of 12 different metrics were identified across these studies. While certain metrics were named differently across the literature, or presented with minor variations in their implementation between scientific studies, these techniques are categorised here based upon their common principles.

### Experimental methodology for evaluation of retained metrics

To assess the metrics’ capability to differentiate distinct inter-joint coordination patterns, a dedicated dataset was generated. This dataset consists of reaching movements executed using varied coordination strategies. Each metric was computed across these diverse datasets, and a comparative analysis was conducted to determine whether the metric outcomes appropriately captured the discrepancies in coordination strategies within the datasets.

#### Experimental setup

To record the different movements, a 4-DOF right arm robotic exoskeleton named ABLE was used [49]. It was designed by the CEA-LIST as a four active DOF robot, with 3-DOF at the shoulder (for abduction/adduction, internal/external rotation, and flexion/extension) and one at the elbow (for flexion/extension). Each joint is composed of an encoder and gravity and friction compensation were coded in the control algorithms, so it can be used as a motion capture device.

A TV screen placed 4 m in front of the participant was used to display targets, and the participant’s end-effector height was also projected on the screen.

**Fig. 1:**
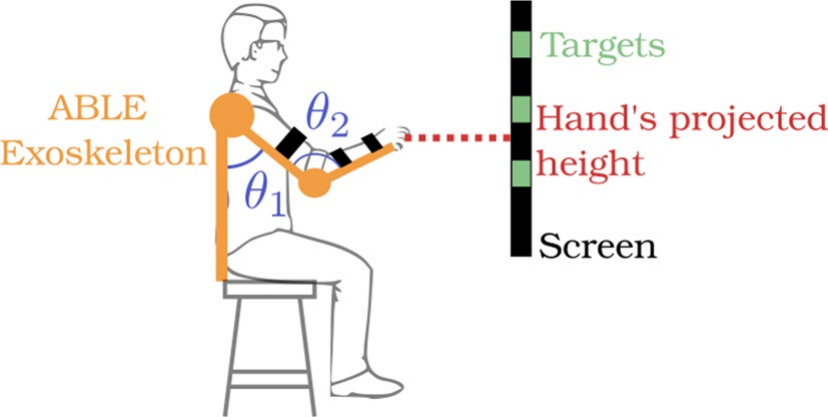
Experimental set up. Participant is wearing the exoskeleton (in yellow) and can use shoulder flexion ($\Theta _1$) and elbow flexion ($\Theta _2$) to reach targets (in green) on the screen in front of him

Three different targets were represented on the screen. The first one was approx. 20 cm above the resting position (hand on the knee of the participant). Each target was placed 30 cm above (along the Z axis) the previous one (Fig. 1).

#### Data collection and processing

**Fig. 2:**
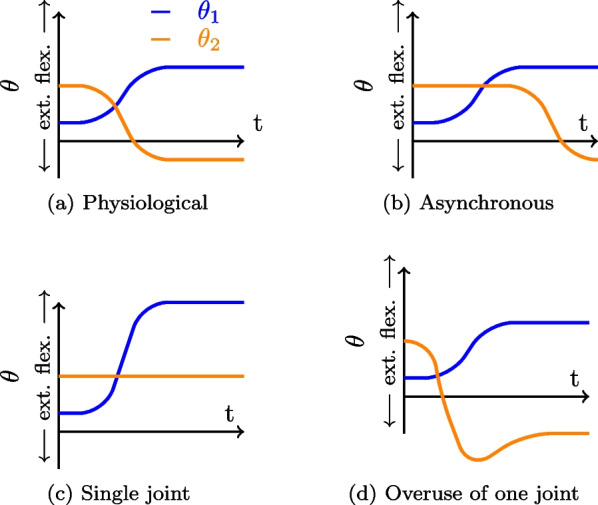
Different strategy of coordination with 2 joints.** a** Physiological coordination,** b** Asynchronous coordination,** c** Single-joint coordination,** d** Overuse of one joint coordination. Shoulder flexion and extension is in blue ($\theta _1$) and elbow flexion and extension is in orange ($\theta _2$)

In order to build a simple dataset, the experimental task involved reaching to a predetermined height (1 Degree of Freedom (DoF) task) using only 2 DoF of the arm (flexion/extension at the shoulder and elbow). The remaining rotational axes of shoulder and elbow joints were immobilised in the exoskeleton while the wrist was fixed using a prefabricated splint. This task was designed such that the number of joints mobilised was reduced whilst still retaining redundant DoF for the specified task. 4 coordination strategies were defined in order to test the different aspects of inter-joint coordination:

- *Physiological* (Fig. 2a), where a participant reached for targets with no specific constraints. This was considered as the baseline coordination strategy.
- *Asynchronous* (Fig. 2b), where the participant was asked to move their joints separately, firstly through shoulder flexion and secondly through elbow extension. This condition was used to test the ability of the different metrics’ ability to characterize temporal coordination.
- *Single joint* (2c) consisted in reaching the target only using one joint, here only shoulder flexion was used.
- *Overuse of one joint* (Fig. 2d) consisted in using one joint excessively. In this case, the shoulder is performing the same movement as the *Physiological* condition, while the elbow is first performing a flexion and then an extension to reach the target. These two last conditions were used to test the ability of metrics’ ability to characterize spatial coordination.

To record the data, one participant was installed in the 4 DoF Able exoskeleton in a transparent mode (gravity balanced) with shoulder abduction and internal rotation locked in a comfortable position for the subject. The screen in front of the subject displayed a slider corresponding to the projection of the subject’s hand height computed directly from the robot’s kinematics. The slider moved in upward and downward directions only (no lateral movement). Rectangles projected onto the screen served as the target, and the starting position was defined as the position where the shoulder flexion angle was 0° and the elbow flexion angle was 90°. For each of the 4 conditions, the participant reached 5 times towards each of the 3 targets.

Joint trajectories were recorded using the encoders integrated into the joints of the robotic exoskeleton. Data processing was conducted using Python. All recorded data were low pass filtered through a 1st-order Butterworth filter (cutoff = 10 Hz). The beginning and end of each movement were defined by 5% of the end-effector maximum velocity. The metrics sourced from the literature were implemented using Python, occasionally utilizing available libraries like statistical libraries. These metrics were computed for each dataset.

This study was approved by the local ethics committee at Sorbonne University and each participant provided informed consent prior to their participation in this study.

### Evaluation criteria

Here we examined the different coordination metrics using a 5-point scale for the following 7 criteria

- *Dimensionality*: How many indicators do we have to analyze?
- *Sensibility*: Is the metric able to distinguish 2 different coordination strategies?
- *Explainability*: Can differences between gestures be explained in a physiological manner using the metric’s output?
- *Computational Simplicity *: How easy is it to implement and compute the metric? A metric based on simple operations such as subtractions, additions, etc. are defined as simple to compute since they are straightforward and give quick results. Metrics that use matrix or statistical calculations are defined as more complex since the result is not straightforward and depending on the size of the dataset and the resources available in the used computer, those calculations can also take some time to output a result.
- *Coordination Pattern*: Does the metric characterize spatial relations?
- *Temporal Coordination*: Does the metric characterize temporal relations?
- *Robustness*: Does the metric provide the same result regardless of task variability? Here, the task’s variability is defined by the variation of the target location.

One last criterion was defined as the *Type of Comparison*. In effect, some metrics aim to extract features from 1 joint trajectory, while others aim to compare 2 or more joints or segments, while others still aim to compare across different conditions. This criterion significantly influences the metric’s dimensionality. To elaborate, metrics that involve pairwise joint comparisons lead to an exponential increase in the number of plots to analyze with the inclusion of more joints. Conversely, metrics designed for condition comparisons often yield more condensed outcomes, encompassing the entirety of movement rather than individual joints. However, this approach can occasionally trade-off in terms of providing a comprehensive explanation.

## Metrics literature review

**Fig. 3:**
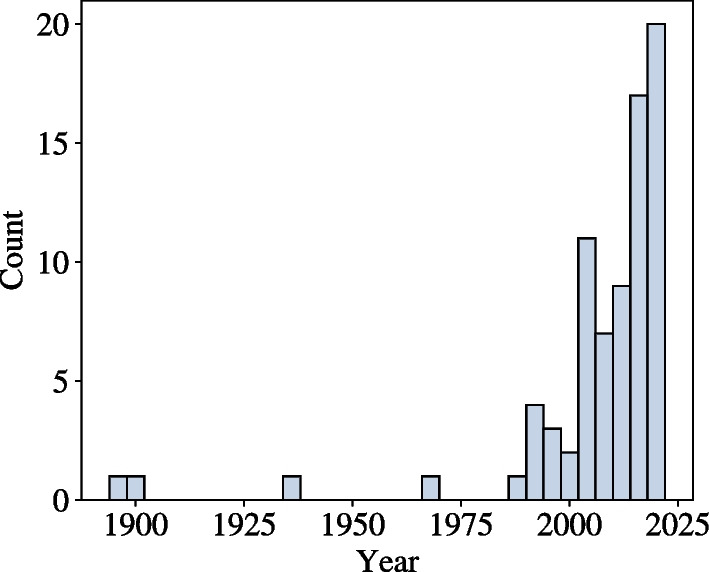
Distribution in time of the articles considered in this review, showing an acceleration of the publication on the subject

**Fig. 4:**
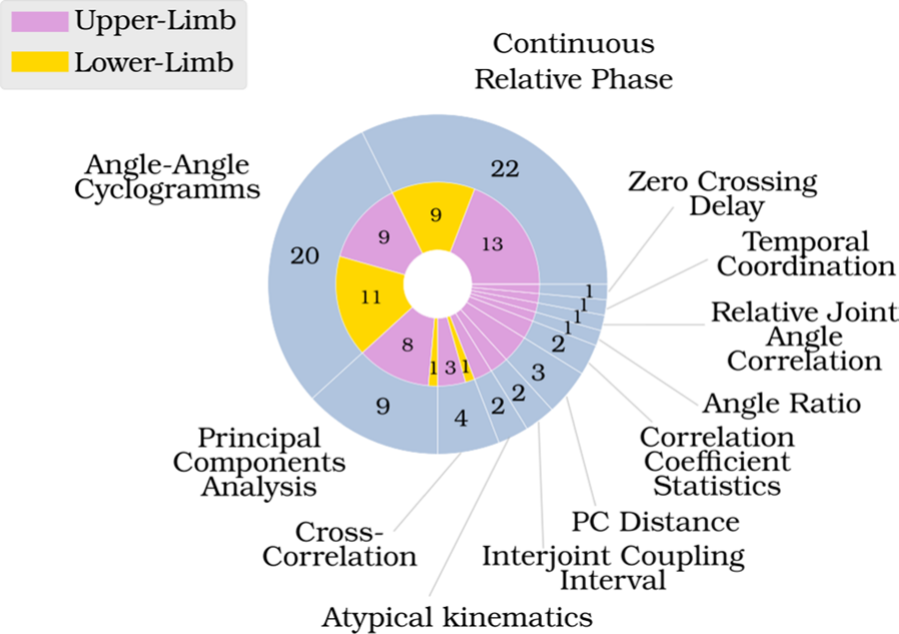
Distribution of the different metrics used in the articles considered in this review

Inter-joint coordination is a topic that has attracted an increasing number of studies in recent years (Fig. 3). When searching for “upper-limb inter-joint coordination” in Google Scholar, 2450 articles were presented, 801 of them were published after 2018 and 319 after 2021. Nevertheless, less than one third of these used metrics based on kinematic data from discrete movements. A total of 12 metrics not including variants were drawn from existing literature and provide diverse methodologies for assessing inter-joint coordination. Certain metrics concentrate solely on particular time events, while others compare the complete trajectories of joints, and others examine overall conditions without delving extensively into joint specifics. This assortment of metrics can potentially emphasize distinct facets of inter-joint coordination. Of the 12 metrics used to measure inter-joint coordination, only two metrics are used in the majority of cases: Angle-Angle Cyclograms and Continuous Relative Phase (Fig. 4).

In the next sections, metrics are presented by order of use, and the following convention is used:

- $\theta \in \mathbb {R}^n$ where n is the degree of freedom represents the joints trajectories,
- $\dot{\theta } \in \mathbb {R}^n$ represents the joints velocities,
- $t \in \mathbb {R}^{0+}$ is the time,
- $\dot{x} \in \mathbb {R}^3$ is the end-effector velocity

Each metric is illustrated with a theoretical example.

### Continuous relative phase

**Definition** CRP, alternatively known as “Temporal Coordination Index” (TCI) [50], is one of the most applied metrics. CRP has been used to quantify inter-joint coordination since 1993 [51], firstly for the lower-limb, and only since the 2000s for the upper-arm [52].CRP extracts the phase angle of the relation between position $\theta$ and velocity $\omega$ for each joint on the overall movement, and then compares the obtained phase angle $\phi$ between different joints. As suggested by [51], “Quantification of inter-joint coordination through the use of the relative phase angle provides information that cannot be obtained through conventional angular position vs time presentation and may lead to substantive differences in interpretation of kinematic data.” CRP transforms the data into a phase plane, enhancing the phase relationship between joints and enabling the determination of which joint takes the lead over the others.

CRP was historically referred to as “Relative Phase Angle” due to its computation involving the disparity between two phase angle signals. The term ”CRP” was introduced as a counterpart to “Discrete Relative Phase” (DRP), which calculates the phase angle between two joints at a specific instant in time, rather than tracking their evolution over the entire movement. DRP has often been used in gait analysis, leveraging readily identifiable time events such as foot placement [53]. However, such events are somewhat more challenging to capture in discrete upper-limb movements, and fail to provide a broader perspective of the entire motion. For these reasons, only CRP is used in this study.

**Fig. 5:**
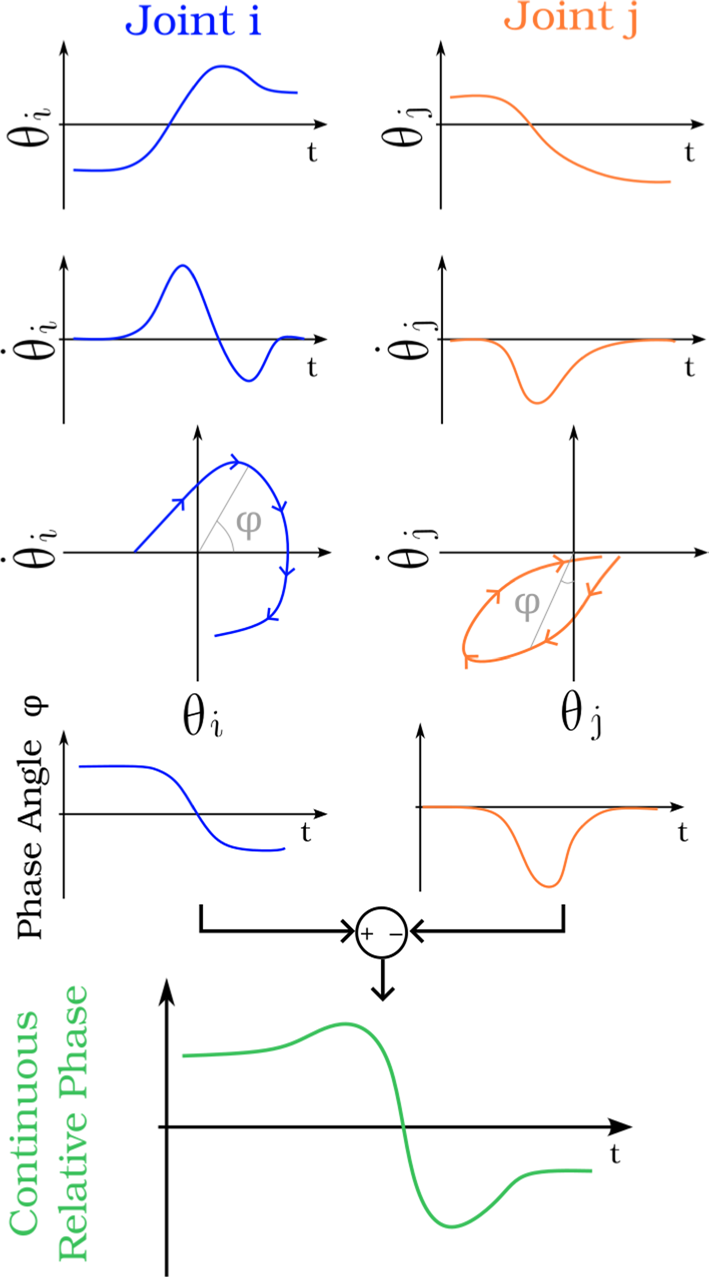
Continuous Relative Phase Computation. For 2 consecutive joints *i* (in blue) and *j* (in orange), position (first row) and velocity (second row) are computed and then plotted together (third row). Phase angles are extracted (fourth row) and subtracted (fifth row)

CRP computation is based on the phase angle, $\phi$ which is the angle between the position/velocity point and the null velocity axis (Fig. 5) of the normalized data.

1 $$\begin{aligned} \phi _i(t) = tan^{-1}\left( \frac{\omega _i(t)}{\theta _i(t)}\right) . \end{aligned}$$

Other techniques such as Hilbert transform can be used on sinusoidal signals to extract the phase angle of a dataset [54].

To compare 2 joints *i* and *j* their respective phase angles are subtracted (Fig. 5) as follows with *i* being the proximal joint, and *j* being the distal joint:

2 $$\begin{aligned} CRP_{i,j}(t) = \phi _i(t) - \phi _j(t), \end{aligned}$$

**Analysis** A constant relative phase means that the relation between the two joints is constant, while a positive or negative CRP means that one joint progressively goes out of phase with respect to the other joint (e.g. a negative CRP means that the proximal joint is lagging behind the distal joint and a positive CRP means that the proximal joint is leading the distal joint).

As CRP is a time series, it can be difficult to visually recognize differences between 2 CRP. In order to simplify CRP analysis, some studies focus on the Mean Absolute Relative Phase (MARP) and Deviation Phase (DP, equivalent to the standard deviation) [55]. It is proposed that if $MARP<30 ^{\circ }$ the joints are moving in phase. On the other hand, if $MARP > 30^{\circ }$ movement is considered as out-of-phase. A small DP indicates that the relation between the 2 joints is stable.

Other articles have suggested different tools to analyze the CRP pattern, such as linear regression [56]. Some variants of the CRP have also been used as Discrete Relative Phase (DRP), to evaluate the timing of key events in each of the angle profiles [53, 57]. However, such methods are more suitable for cyclic tasks since they compares the temporal dispersion of events.

### Angle–angle plot or cyclograms

**Fig. 6:**
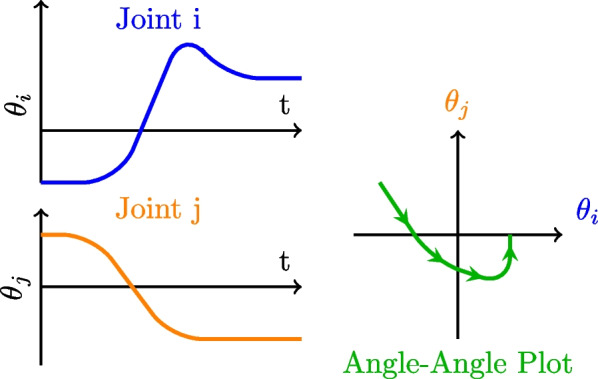
Angle-Angle Plot or Cyclogram. Two joints’ position (blue and orange) are plotted together (in green)

**Definition** The angle-angle plot is one of the oldest inter-joint coordination metrics [58]. Also named angular covariation plots [59] or cyclograms [60]. Angle-angle plots are 2D or 3D plots [61] where each axis of the plot is one joint’s position or velocity (Fig. 6). Angle-angle plots emphasis the relative trends in angular displacement for each joint over time [58]. By exposing those relative patterns, alterations in inter-joint coordination can be visually discerned. In certain instances, polar angle plots have also been employed to appraise joint coordinations [62].

**Analysis** If a coordination exists between the 2 variables, a relation should appear as a distribution of data points around the diagonal line or plane of the plot.

This simple representation for coordination has been widely used, but characterizing (geometrically) the coordination pattern can be quite difficult due to the large number of curves to analyze (number of curves grows quadratically ($n^2$)). In order to simplify the analysis of those plots, different characteristics can be extracted as: the global slope [63], the mean magnitude (mean distance between two consecutive points [56]), angular coefficient of correspondence (slope of two consecutive points [64, 65]) etc. Those measurements are also used as inter-joints coordination metrics.

### Principal component analysis

**Definition** Principal Components Analysis (PCA) is a method used to reduce dimensionality of a dataset. First developed in the early 20th century [66, 67], the goal is to extract the main components of a multidimensional dataset, so it becomes easier to interpret since only independent features are left. In human movement analysis, PCA is used to extract the main independent relations between a set of joint trajectories.

PCA is computed on position or velocity-centered data. From the covariance matrix of the dataset, eigenvalues and the corresponding eigenvectors are extracted. Each eigenvalue represents the amount of variance of its corresponding component, while the corresponding eigenvector indicates how much each variable is contributing to the component. PCA can be computed both from the correlation matrix and from the covariance matrix. However, computing PCA on non-standardized data using the covariance matrix will remove data with a smaller range since they have less variability than data with a greater range. Alternatively, using the correlation matrix is equivalent to directly standardising the dataset (mean data is set to 0 and the standard deviation is set to 1).

Extended versions of PCA such as functional Principal Components Analysis (fPCA) have also been explored [68] in order to consider temporal variation in the movement (and not only postures, as is typically the case in PCA). Sparse Principal Component (SPCA) [69] is yet another variation of the PCA method that sets to 0 the variance of the variable that has a small variance, so they are not disturbing the interpretation of the main components. Since PCA is a global method, it doesn’t require precise joint-angle measurements to be able to extract main components of the coordination strategy used. For example, [70] directly used the 3D coordinates of markers placed on the human body during walking as inputs.

**Fig. 7:**
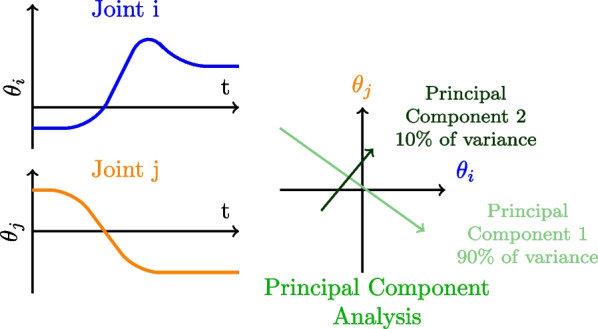
Principal Component Analysis. Two joint positions (in blue and orange) are plotted together (in gray) in order to find a new vector basis (in green) that maximizes the variance of the dataset

**Analysis** PCA aims at extracting a common pattern in a dataset. The first step in the analysis of PCA results is to examine the percentage of variance explained by each Principal component (PC). The first component of the PCA represents the dimension of the data that has the largest possible variance in the data set, whereas the last components represent the data that have the smaller variance. In the example Fig. 7, the first PC accounts for 90% of the variance. Then, inside each PC, the weight of each original variable can be examined [20]. Here, the first PC is mainly composed of the position of the *i*th joint. Other PC analysis techniques have been developed. [71] has reconstructed the PC evolution over time by summing all the variables weighted by their PC results. This PC evolution has been analyzed in [72], using a hierarchical classification algorithm to detect changes in coordination. However, this last method works with a dataset where the first PC contains more than 90% of the dataset variance. When the first PC represents less variance, the analysis must be extended to the other PC, increasing the dimensionality of the results.

When comparing different PCA results, one hypothesis is to say that the alteration of inter-joint coordination leads to different joint contributions. A loss of inter-joint coordination leads to a higher number of PCs to explain the variability of the movement, since joints are uncoupled. Another indicator to check is the weight of each variable inside each PC. Indeed, a loss of inter-joint coordination leads to more simple and less variable contributions. For example, fewer PCs are needed to reconstruct the movements of stroke patients than non-stroke patients [68].

One main point of attention when using PCA is the size of the dataset. Indeed, when a too small dataset is used, the results obtained with the PCA are unstable. There is no ’minimal size’ for a dataset on PCA, one way to verify that PCA can effectively be used in this case is to bootstrap or cross-validate the PCA by deleting or exchanging some data in the dataset and computing the PCA again. If the result is similar to the result obtained with the original dataset, the result is stable, PCA can be effectively used in this case. If results vary a lot with the modification of the dataset, the result is unstable and should be used cautiously.

### Cross correlation

**Definition** Cross Correlation is a signal processing method used to measure similarity of two series as a function of the displacement of one relative to the other (sliding dot product). When cross-correlation is used to measure coordination, usually only a zero time lag is considered [73], however recent studies have begun to explore the result of cross-correlation with different lags [74]. Cross-correlation is a metric that can be used on different types of temporal signals, for example [75] have computed cross-correlation based on CRP signals.

3 $$\begin{aligned} corr_{\theta _i,\theta _j}(k) = \sum _{m=-\infty }^{\infty } \theta _i(m) \theta _j(k-m). \end{aligned}$$

**Analysis** The higher the cross-correlation is, the more the 2 joints are coordinated since their signal is considered similar. However, in case of a large difference in movement amplitude, the cross-correlation can be high even if the joints are poorly coordinated. Joint position may be normalized to avoid this artifact.

### Atypical kinematics

**Definition** Atypical kinematics is a metric suggested by [76]. The goal of atypical kinematics is to extract portions of time normalized movement sets where joints trajectories differ from a reference movement using a filter based on PCA. The result gives a normalized time period and the joint sets for which the trajectories differed most.

**Fig. 8:**
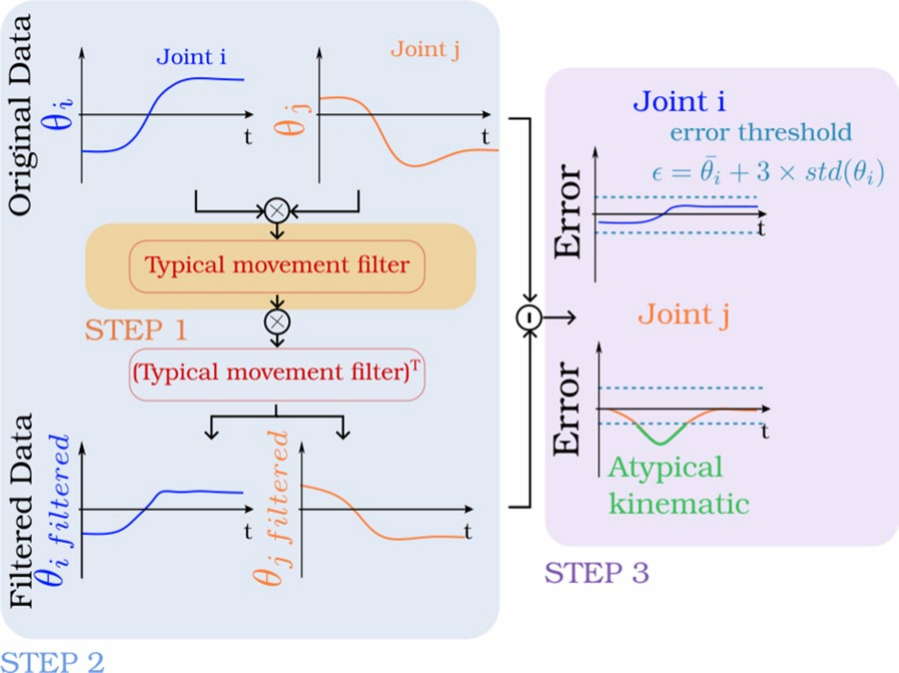
Atypical Kinematics. First Step (in orange), compute typical movement filter. Second step (in blue), filter the data. Third step (in purple), compute the error between the original and the filtered data

Atypical kinematics needs to be computed in three distinct steps. The first step is to build a typical movement filter. The typical movement filter is built from the principal components extracted for each percent of the reference movement (from 0% to 100%). The second step is to filter all the data using this filter. To achieve this second step, each percent of the data to analyze are multiplied by the corresponding typical movement filter and then by the transposed of this filter. Finally, the last step is to define which part of the moment is atypical or not. To this purpose, error between the original and filtered data is computed for each percent of the movement. If the error is greater than 3 times the standard deviation of the reference movement, the movement is classified as atypical for this time period (Fig. 8).

**Analysis** The more atypical kinematics moments there are, the less the movement is coordinated [77]. The same considerations regarding the size of the dataset in PCA should be applied for atypical kinematics.

### Inter-joint coupling interval

**Definition** Inter-joint Coupling Interval (ICI) is a temporal metric used by [78]. This metric highlights relations between the end of the activation for each joint, also named settling time. This temporal delay provides insight into the sequence of joint deactivation and the duration for which one joint remains active after the cessation of movement in another. As is the case with many temporal metrics, ICI doesn’t provide comprehensive inter-joint coordination information spanning the entire movement but rather focuses solely on the distinct event of joint deactivation.

**Fig. 9:**
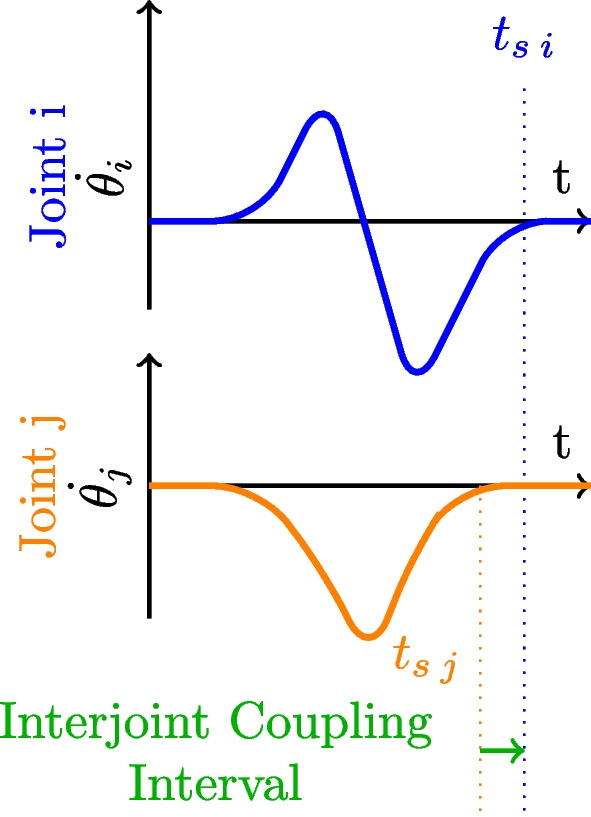
Inter-joint Coupling Interval. Inter-joint coupling interval is the delay between 2 joint’s settling ($t_s$)

The joint’s deactivation is defined as the moment where the joint’s velocity is lower than 5% of its maximum absolute velocity. For each pair of joints, their respective end of deactivation time is subtracted (Fig. 9).

4 $$\begin{aligned} ICI_{i,j} = t_{s \,j} - t_{s\,i}. \end{aligned}$$

**Analysis** If ICI is close to zero, and the standard deviation of ICI for all movement is low, that means that the 2 joints are well coordinated. The higher the ICI value is, and the higher the standard deviation of ICI is, the less the joints are coordinated in a temporal manner.

It should be noted that even for healthy subjects, the average ICI might be slightly lower than zero, as a proximal-to-distal sequence of joint activation is generally observed [28] in routine tasks.

### Distance between PC

**Fig. 10:**
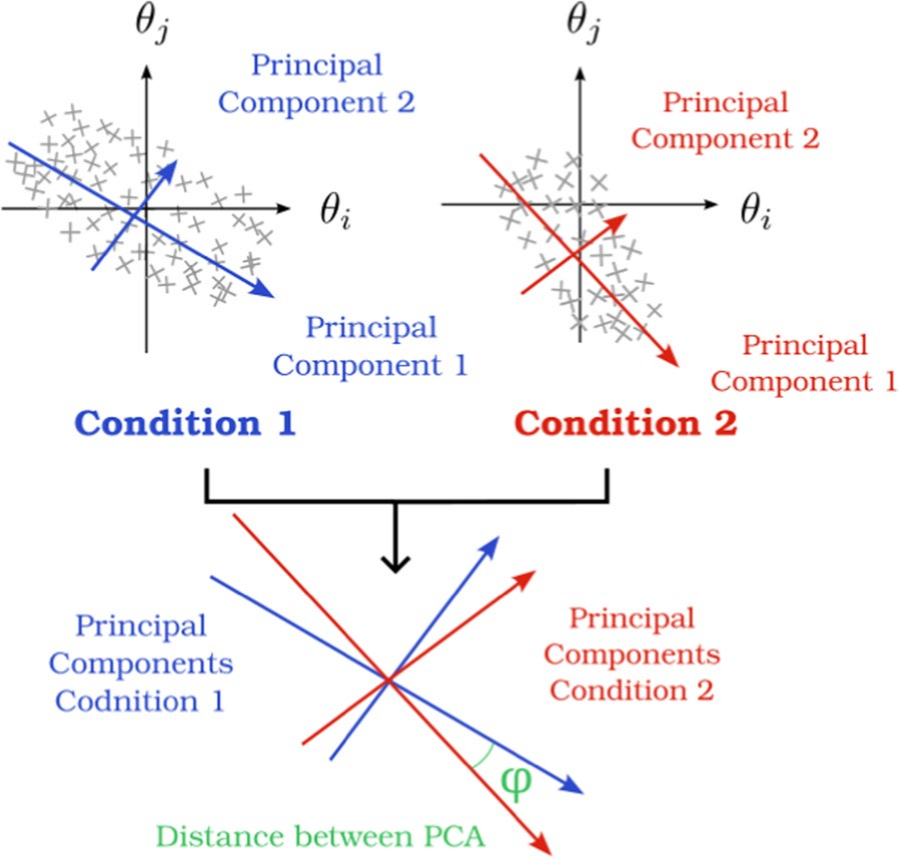
PCA distance. The distance between 2 subspaces defined by principal component analysis (in blue and red) is defined by the angle $\Phi$ between the 2 spaces

**Definition** This metric, developed by [79], computes the angle between subspaces defined by Principal Components Analysis of the kinematic data obtained in 2 different conditions. The result of this metric quantifies the extent of disparity between two coordination strategies, rated on a scale from 0 to 1. However, details regarding this disparity are somewhat limited, as the specific joints with differing trajectories or the precise segment of movement exhibiting differences is not identified (Fig. 10).

Let U and V $\in \mathbb {R}^{n}$ be 2 subspaces defined by PCs of condition 1 and condition 2. The distance between U and V is defined as

5 $$\begin{aligned} dist(U,V) = \sqrt{1-s_{min}^2(U^TV)}. \end{aligned}$$

With U and V being a set of unit vectors defining a subspace and $s_{min}$ the minimal singular value of $U^TV$.

**Analysis** The higher the distance is, the more different the two coordination strategies are [80]. However, this metric does not tell which joint of the coordination or part of the movement is coordinated differently.

### Correlation coefficient statistics

**Definition** The Pearson Correlation Coefficient [81] or Spearman Rank Coefficient [82] are statistical tools used to evaluate the strength of a linear or monotonic relation between two variables. The correlation between two joints offers insights into the extent to which these joints exhibit coordinated behavior, indicating whether they evolve together in a linear or monotonic manner. The result ranges between $-1$ and $+1$ and a p-value is associated to the result. A small p-value indicates that a significant correlation exists between the 2 variables.

**Analysis**

The closer the Correlation Coefficient is to 1 or $-1$, the stronger the linear relationship between the two joint angles. This result can be used only if the corresponding p-value is lower than 0.05 (5% significance level). If data are not filtered, strong correlation can emerge from noise inherent to the signals. A strong correlation can also be found if one joint moves only to a limited degree (due to the movement of the other joints or micro-movements for example), even if this behaviour might not fulfil the qualities expected of “coordinated movement”.

### Angle ratio

**Definition** The Angle Ratio is another relatively simple measure that takes 2 joint trajectories, and for each timestamp computes the ratio of both angles. To simplify, only the ratio at the end-effector maximum velocity can be computed [83]. The angle ratio reveals which joint within a pair contributes the most to the overall movement. Yet, this metric’s accuracy might be influenced by joints with varying overall ranges. As an illustration, the wrist commonly exhibits a smaller angle measurement compared to the shoulder.

6 $$\begin{aligned} AR_{i,j}(t) = \frac{\theta _i(t)}{\theta _j(t)}. \end{aligned}$$

**Analysis** If the range of motion of the 2 joints are different (i.e, wrist and shoulder), the data should be normalized at their range before computing the angle ratio. Between 2 conditions, if the joint angle ratio is increasing, that means that joint *i* is making a greater contribution to the movement. On the contrary, if the joint angle ratio decreases between 2 conditions that means that joint *j* is contributing the most.

### Relative joint angle correlation

**Definition** Relative Joint Angle Correlation is a metric presented by [84] based on the analysis of the covariance matrix between 2 relative joint angles. Relative joint angles are calculated from the segment’s vector, this indicates that the kinematic parametrization of the chain used to extract joint angles (i.e, International Society of Biomechanics Convention for upper-limb [85], robot kinematic chain, etc.) does not affect the result. This metric in an indirect measure of inter-joint coordination based on kinematic data.

RJAC is a measurement of inter-limb synergy, and by extension the inter-limb synergies reflect the inter-joint coordination strategy. Notably, RJAC values have been demonstrated to exhibit correlation with the Fugl-Meyer Assessment of Sensorimotor Recovery After Stroke for Upper-Limb Extremity (FMA-UE) [84] which gauges motor abilities and joint amplitude. This association underscores how RJAC not only reflects motor skills but also provides insights into joint coordination.

**Fig. 11:**
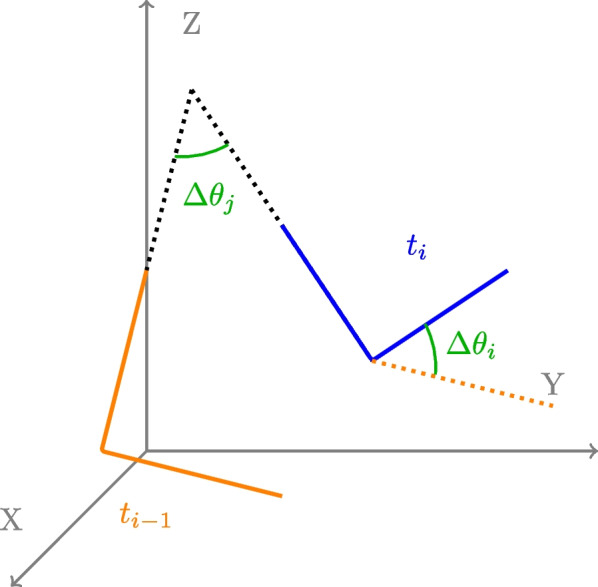
Relative Joint Angle Correlation. RJAC is based on joint’s position $\Delta$ between consecutive timestamp

Let *i* be the proximal segment and *j* the distal segment, defined as 3D vectors for each *t* timestamp (Fig. 11).

$$\Delta \theta _j(t) = arccos\left(\frac{\textbf{i}(t). \textbf{j}(t)}{|\textbf{i}(t)||\textbf{j}(t)|}\right) - arccos\left(\frac{\textbf{i}(t-1). \textbf{j}(t-1)}{|\textbf{i}(t-1)||\textbf{j}(t-1)|}\right)$$.

For the first joint, where there is no proximal segment, the relative angle is computed as follows:

$$\Delta \theta _i (t) = arccos\left(\frac{\textbf{i}(t-1). \textbf{i}(t)}{|\mathbf {i(t-1)}||\textbf{i}(t)|}\right)$$.

Then RJAC is computed as:

$$RJAC(i,j) = \frac{C(\Delta \theta _i, \Delta \theta _j)}{\sqrt{C(\Delta \theta _i, \Delta \theta _i)C(\Delta \theta _j, \Delta \theta _j)}}$$.

**Analysis** To compare different datasets together, the absolute value of the RJAC matrix is considered. A lower value of the RJAC’s matrix determinant means that joints are less coordinated. Similar to other metrics that give only one final value for a whole condition, this metric provides insight into how different two datasets are. It does not provide insight on the origin of the difference.

### Temporal coordination

**Definition** Temporal Coordination is used by [86] and focuses on determining the delay between the activation time of the joints compared to the beginning of the movement. Similar to the inter-joint coupling interval (ICI) metric, this measure focuses on a single time delay between two specific time events. Here, it emphasizes the point at which a joint begins to engage in the movement. Among individuals without impairment, this delay is typically minimal and follows a proximal to distal sequence, indicating that proximal joints usually initiate movement before distal joints, showing effective coordination across all joints.

**Fig. 12:**
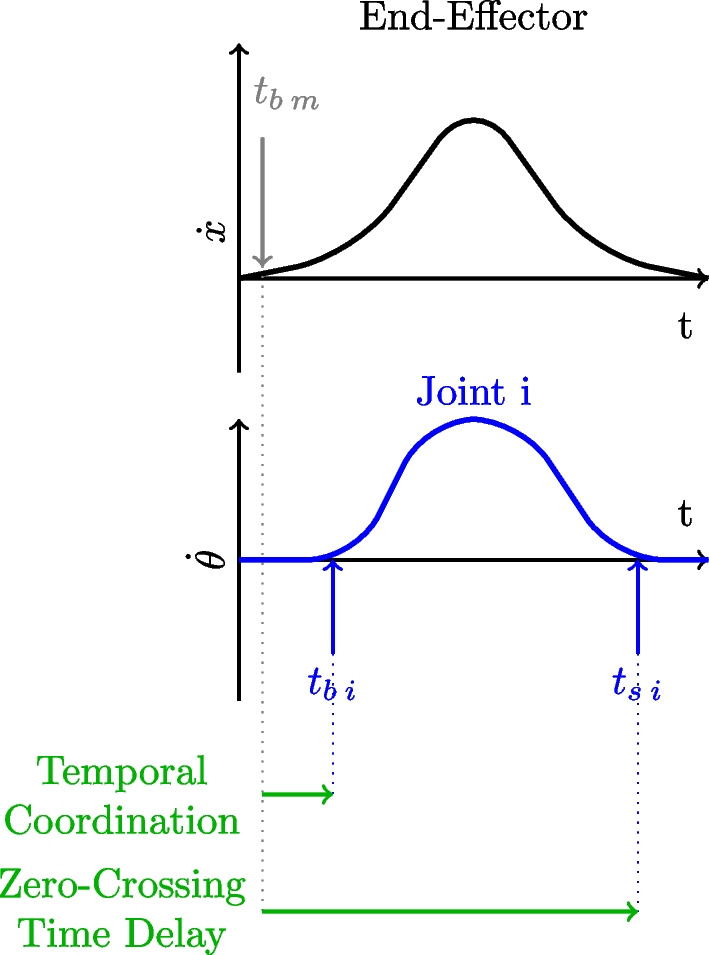
Temporal Coordination and Zero Crossing Time Interval. Temporal Coordination is the delay between the beginning of the movement ($t_{b \, m}$) and the start of the joint ($t_{b \, i}$). Zero-Crossing Time Delay is the delay between the start of the movement and the end of activation of the joint ($t_{s \, i}$)

Joint activation is defined as the instant when joint velocity is greater than 5% of its maximum absolute velocity (Fig. 12).

7 $$\begin{aligned} TC(i) = t_{b \, i}-t_{b \, m}. \end{aligned}$$

**Analysis** As per the ICI, the smaller the TC is, the more the joints are coordinated from a temporal point of view.

### Zero crossing time interval

**Definition** Zero-Crossing Time Interval is a metric that computes the time between the beginning of the movement and the deactivation of the joint [87]. It is also a temporal metric, focusing exclusively on the temporal synchronization between the completion of joint angle variation and the beginning of the entire movement. Zero-Crossing Time Interval quantifies how extended or delayed a joint’s involvement is in the overall movement. However, this metric does not provide insight into the distinction between the activation time and the duration of activation. To elucidate this aspect, a comparison with the Temporal Coordination metric is necessary.

The Joint’s deactivation corresponds to the instant when the joint’s velocity becomes lower than 5% of its absolute maximal velocity (Fig. 12). Then each Zero-Crossing Time Interval can be compared for different conditions.

8 $$\begin{aligned} ZC(i) = t_{s \, i}-t_{b \, m}. \end{aligned}$$

**Analysis** If the Zero Crossing Time Interval is similar over different conditions, that means that the coordination is similar in different conditions.

## Experimental results

### Quantitative comparison of the metrics

**Fig. 13:**
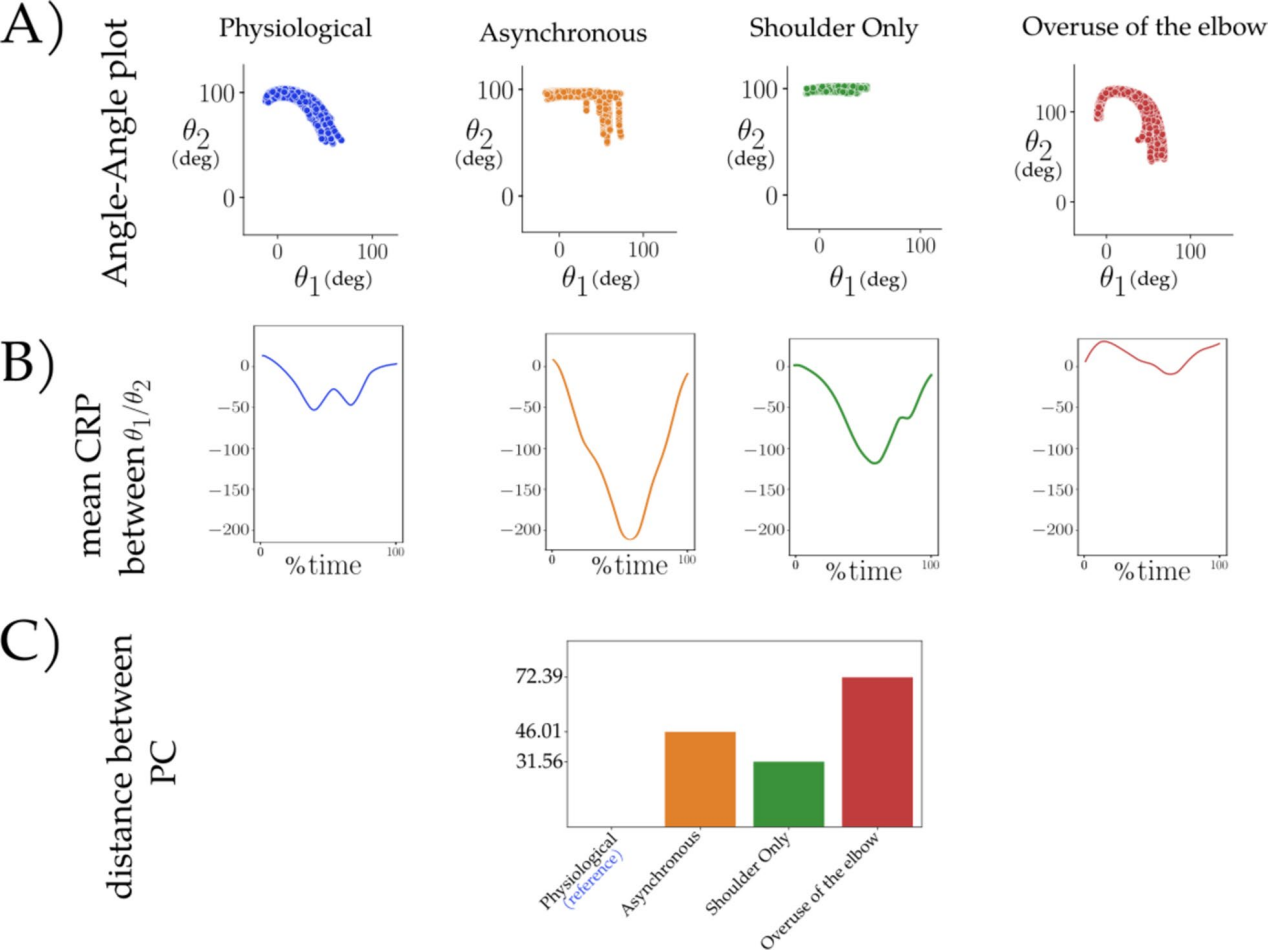
Typical graphics obtained for the 4 conditions for 3 inter-joint coordination metrics, reduced to the observation of 2 DoF only: shoulder flexion as $\theta _1$ and elbow flexion as $\theta _2$. **A** Angle-angle plot. **B** Mean CRP curves, C) distance between PC subspace with the Physiological condition as reference

All metrics shown were computed based upon the datasets derived from the four different coordination strategies described previously. Figure 13 displays typical graphs for three metrics: angle-angle, CRP, and PCA distance, across the four conditions (each represented by a distinct color). For these metrics, only visual analysis was conducted to distinguish between conditions, as conducting statistical analysis would require extracting specific features (such as the mean slope of linear regressions for the angle-angle plot or the mean CRP), or involving more subjects per condition (to calculate the distance between PC for each subject in each condition and perform statistical analysis on the entire group).

All metrics presented above have been computed on the 4 different coordination strategies datasets. Figure 13 presents typical graphics obtained for 3 metrics : angle-angle plots, CRP and PCA distance, for the 4 conditions (one color per condition). For the presented metrics, only a visual analysis has been done to differentiate the conditions and no statistical tests were carried out since the size of the illustrative dataset was limited. The results obtained have been summarized in the Table 1.

To simplify the overall analysis, a scale composed of 5 colors was used to evaluate the metrics. For all criteria (except dimensionality) the color was attributed based on the ability of the metric to differentiate between conditions.The green color is used when the result of the metric for a specific criterion is good, whereas the dark red color is used when the metric is not meeting the conditions for a criterion.

Table 1 presents the different results. In this table, metrics have been categorized by their *type of comparisons*. This categorization approach was adopted due to its direct influence on the dimensionality and the level of explainability needed. The first 2 metrics analyze each joint trajectory separately, resulting in metrics with relatively greater dimensionality. The second part of the table presents metrics that compare joints on a pairwise basis, which implies greater dimensionality. The different results in dimensionality for this group of metrics are due to metrics that also vary temporally or metrics that give results for the whole movement. The third group of metrics is composed of only one metric: Relative Joint Angle Correlation. This metric compares segments rather than joint angles on a pairwise basis, leading to fewer indicators to analyze since for the human arm (not considering the wrist), 4 joints angles are necessary to describe movement whereas the human arm is only composed of 2 segments (arm and forearm). Finally, the last group is composed of those metrics which evaluate a whole condition. These metrics are all based on PCA. Additionally, classifying metrics depending on the temporal or spatial analysis was rather complex since, for some metrics, it depends on the input data (i.e, position or velocity data).

**Table 1:**
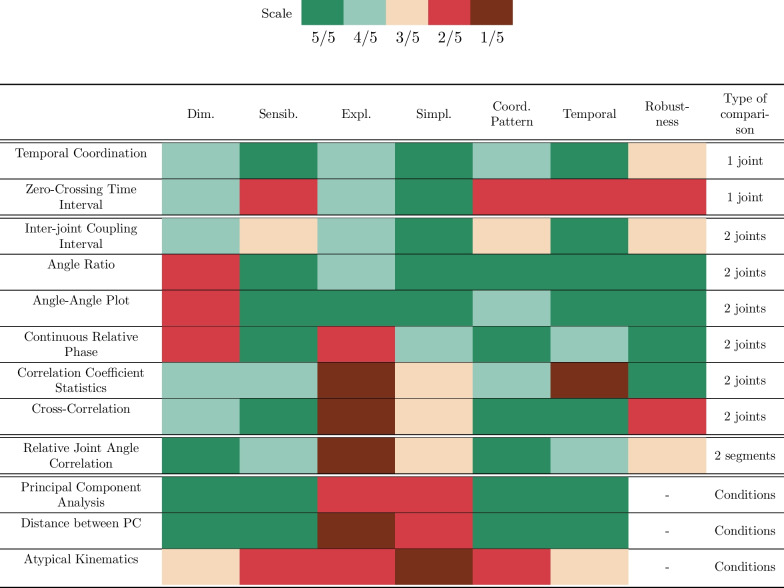
Summary of inter-joints coordination’s metrics

Robustness can’t be measured on metrics using Principal Components Analysis, since these methods needs a large amount of data to get a meaningful result. To compute PCA, the overall dataset has been considered, not splitting data by target height, so robustness was not tested.

### Detailed quantitative and qualitative analysis of the metrics

**Time delay metrics** Of the 12 metrics examined, 3 are based entirely on time delays between different events (Temporal Coordination, Zero-Crossing Time Interval and Inter-joint Coupling Interval). Those metrics are easy to implement since they are based on subtractions and their interpretability is straightforward since the direction and extent of the delay offer direct insights. Nevertheless, these metrics are only capable of capturing a fraction of the temporal dimension of inter-joint coordination.

Temporal coordination enhances when a joint starts participating in the movement with respect to the beginning of the end-effector movement. Given that each joint’s starting time is considered, numerous Temporal Coordination results are generated - equivalent to the number of joints. This metric proves efficient at distinguishing the four coordination strategies if data of the exact same targets are compared. However, it can only explain one event within the overall movement. It’s important to note that this metric’s outcome varies significantly based on target positions, rendering it sensitive and unreliable when target positions change substantially across repetitions.

Zero-Crossing Time delay, on the other hand, highlights when a joint stops participating in the movement, with respect to the beginning of the movement. Just as with Temporal Coordination, there’s one distinct time delay for each joint. However, this metric is highly sensitive to minor movements and yields identical outcomes for the *Physiological* and *One joint only* coordination strategies, as well as for the *Asynchronous* and *Overuse of a joint* coordination strategies. Similar to the previous metric, it’s considerably influenced by target positions, making it valuable only when joint deactivation is the focal point of the study.

Finally, inter-joint coupling interval enhances the delay between the end of use of two joints. The dimensionality of this metric is higher since it compares the joints pairwise. While it can differentiate some coordination strategies, it doesn’t capture the disparity in all cases, such as the similarity in results between the *Physiological* and *Over use of one joint* strategies.

Yet, the three presented metrics provide no information on how the joint’s trajectory evolves around these events. They are also easily influenced by micro-movements, leading to the conclusion that joints are coordinated even if one joint’s participation to the movement is negligible. Among those three temporal metrics, the most sensible and robust one appears to be the Temporal Coordination.

**Joint trajectories comparison metrics** Two metrics are based on direct comparison of joint trajectories, either by computing a ratio such as Angle ratio or by plotting them against each other, as is the case for the Angle-Angle plot. These metrics are efficient to distinguish both spatial and temporal coordination. Yet, their major problem is the dimensionality. These metrics are computed for each possible pair of joints, that means that for *n* joints, there are $\frac{n!}{2!(n-2)!}$ indicators to analyze. When processing data with more than 3 degrees of freedom, it becomes difficult to obtain sufficient perspective regarding the coordination strategy. Both metrics are great at differentiating the four coordination strategies and are really simple to compute. The angle ratio involves a somewhat trickier connection to physiological processes, as it necessitates converting the ratio back into joint behavior. However, the angle-angle plot gives results that are visually comparable between the natural and the overuse of the elbow condition. To discern distinct coordination strategies more accurately, supplementary indicators or tools like plot area coverage or linear regression slope are imperative. Furthermore, incorporating additional measures like the global slope not only aids in reducing dimensionality but also condenses temporal series data into a singular value.

**Metrics combining position and velocity** Continuous Relative Phase is the only metric based on both position and velocity data. This metric can differentiate visually the 4 coordination strategies, even though characterizing CRP curves using Mean Angular Relative Phase (MARP) or Deviation of Relative Phase (DRP) could be advantageous in instances where CRP curves exhibit visual similarity. For instance, in our dataset, CRP generates outcomes that lack pronounced visual distinctions between the *physiological* and *asynchronous* conditions. Nevertheless, the introduction of MARP computation renders the differentiation between these two conditions more apparent. Furthermore, the mix of position and velocity makes it difficult when attempting to extrapolate into physiological terms.

**Statistics** Another group of metrics are the metrics based on statistics such as the Pearson Correlation Coefficient, Cross Correlation or Relative Joint Angle Correlation. They all have the disadvantages to be difficult to explain. Since they are based on complex calculations, the physiological implications may be more difficult to appreciate. The insights gleaned from these metrics result in a binary analysis, categorizing the relationship between two joints or segments as either correlated or not. Nevertheless, their implementation is often straightforward, as many data processing tools provide pre-coded functions within their toolboxes to compute these correlations. It’s important to note that correlation coefficients, such as Pearson’s, lack the ability to differentiate between coordination strategies that evolve over time.

**Metrics based on principal component analysis** The last 3 metrics are based on principal component decomposition. These metrics offer the key benefit of reduced dimensionality, facilitating the differentiation between various datasets. However, explaining the disparity between two movements using only PCA outcomes can be challenging. Metrics based on PCA also require a large amount of data and present complexity in computation, particularly in a crucial step: determining the requisite number of principal components, which depends on the dataset’s variance, the task’s dimension ect.

The Atypical Kinematics metric further amplifies complexity due to its multi-step process involving the construction of a filter based on PCA results and subsequent multiplication of datasets by this filter. In our dataset, encompassing a sole subject with five repetitions for each of the three targets, the atypical kinematics metric failed to yield dependable outcomes. This metric necessitates a larger dataset for accurate application (more than five repetitions per target) to derive meaningful averages and standard deviations essential for correctly calculating the error threshold.

In terms of interpretability, metrics like the distance between principal components offer a direct means to differentiate coordination strategies. However, the resulting value ranging between 0 and 1 provides limited insight into the physiological aspects of the movement.

The Distance between PCs metric affords an initial overview of the movements in question. Subsequently, delving into PCA and assessing the significance of each variable’s weight within the principal components can provide a pathway to gaining deeper insights into the movement.

## Discussion

In conclusion, the selection of an appropriate inter-joint coordination metric remains a challenge, given the absence of a universally flawless metric. The choice depends on the specific attributes to be emphasized, the interpretation of inter-joint coordination, and the metric’s ease of implementation. As a result, one or a combination of these approaches may prove suitable based on the given context. Answering the 4 following questions can lead to the best metric(s) to use:

- Is the spatial or the temporal coordination the main feature to enhance?
- Is it important to be able to tell exactly what’s happening in the movement at the anatomical level, or is a global assessment sufficient?
- How fast do we need the metric to be computed?
- How large is the dataset?

Guided by the responses to these four questions, one or multiple metrics can be selected. For instance, if the study primarily revolves around the temporal dimension of coordination, metrics scoring 4/5 or higher in the Temporal category should be prioritized. Subsequently, if achieving a comprehensive understanding is crucial within the given context—perhaps for tailoring a more precise rehabilitation treatment plan—metrics with an explainability score of 4/5 or greater should be retained. Depending on the available computational resources, a decision can be made to retain metrics that demonstrate a high level of simplicity (with a high Simplicity score). Lastly, metrics reliant on PCA might be disregarded if the dataset is limited in size (with only a few repetitions of movements).

To provide concrete illustrations, let’s consider two distinct contexts: one related to rehabilitation, specifically post-stroke recovery, and the other focused on experimental studies involving upper-limb exoskeletons. In the rehabilitation context, particularly after a stroke, both spatial and temporal coordination play vital roles. However, spatial coordination may receive relatively more emphasis, as one of the primary rehabilitation goals is to restore functional range of motion. In rehabilitation processes, it is crucial for metrics to offer a physiological interpretation, be of simple use, and yield prompt results, considering that patients are waiting for guidance. Moreover, the datasets tend to be relatively small (individual datasets), comprising only a few repetitions of specific movements. In this scenario, the most suitable metrics include the Angle-Angle plot and Temporal Coordination. On the other hand, in an experimental study involving exoskeletons, both temporal and spatial aspects of coordination hold equal importance. The need for providing a physiological explanation for the movements is not as critical as in the rehabilitation setting. Additionally, there are usually no strict constraints on the computational speed or ease of metric implementation, since all processing occurs offline after the experiment. Furthermore, the datasets are typically larger, containing numerous repetitions of the same movements for multiple participants. In this case, the most relevant metrics include CRP, PCA or the distance between Principal Components.

Once the pertinent metrics have been identified, the implementation process requires careful consideration. As each metric can be computed in distinct ways, Table 2 outlines practical recommendations for each metric, aiming to extract the most significant results from each one.

**Table 2:** Recommendations for the use of metrics

**Metrics**	**Recommendations**
Temporal coordination	–
Zero-crossing time interval	–
Inter-joint coupling interval	–
Angle ratio	–
Angle–angle plot	$\bullet$ Same limits on axis to avoid noise zooming
	$\bullet$ Using position data enhances better spatial coordination strategy
	$\bullet$ Using velocity data erases differences due to different starting positions
	$\bullet$ The ratio of the widths of the point distributions highlights the coordination pattern strategy
	$\bullet$ The area covered by the point shows the temporal coordination strategy
	$\bullet$ Coupling this metric with other indicators (as the Angular Coefficient of Correspondence for example) helps to reduce its dimensionality
Continuous relative phase	$\bullet$ Normalize data at the range
	$\bullet$ Unwrap the result to get a meaningful MARP
Correlation coefficient statistics	$\bullet$ Compute with data position
	$\bullet$ Always check the p-value, if too high, the result is not interpretable
	$\bullet$ Spearman or Pearson Correlation Coefficient
	$\bullet$ Use a low-pass filter on data first
	$\bullet$ Use a threshold on data’s velocity to remove micro-movement (if the joint velocity is below the threshold, joint trajectory is considered constant)
Cross-correlation	$\bullet$ To compute on velocity data
	Really sensitive to targets’ position
Relative joint angle correlation	–
Principal component analysis	$\bullet$ To compute on velocity data
Distance between PC	$\bullet$ To compute on velocity data
Atypical kinematics	$\bullet$ To compute on velocity data
	$\bullet$ Needs a very large amount of data

## Limitation and perspective

Given the different metrics and results obtained by testing them, a few points can be underlined.

### Limitations of present study

The methodology used for this study presents some limitations.

**Data collection methods** A first point to discuss is the method used to collect data. The data set used was composed of simulated behaviors that are not representative of daily-life movements. This was done in order to enhance each metric’s specificity separately in order, not to build a classification of the metrics, but to be able to choose cleverly the metric needed depending on the experiment and the hypotheses.

**Simple dataset** One of the main limitations of the results presented here is the simplicity of the dataset used. Indeed, the dataset was composed of movement with 2 DoF achieving a 1 DoF task. This is not representative of upper limb coordination in everyday life conditions where, on a segmental level, the human arm mobilises 7 DoF. In addition, the 2 DoF arm to 1 DoF task only allows 1 DoF redundancy, which limits the overall variability of the movement dataset.

Another means of increasing the dataset’s variability would have been to introduce noise so as to test the different metrics’ robustness.

Finally, the dataset was built upon 1 subject’s movements only. Building a dataset with more subjects would have introduced more variability in the data. This dataset was a first step into evaluating and understanding the different coordination metrics, but to build more reliable conclusions, the analyzed metrics should be tested on a dataset with more variability (number of DoFs and participants).

### Perspective

**Considering the task** None of the metrics described here account for inherent task constraints. Unlike the Uncontrolled Manifold (UCM), the task’s goal is never specified in the listed metrics. In the UCM, joint velocities are separated into the controlled task-space and the uncontrolled null-pace. The controlled task space is necessary to complete the task, while the null-space contains joint velocities that contribute to the joint variability in the task (i.e, the different coordination strategies) without affecting the end-effector trajectory (i.e, in reaching tasks, the swivel angle is usually in the null-space). Separating the *controlled spaces* and *uncontrolled spaces* simplifies the problem and gives a theoretical framework to explain how the central nervous system deals with multiple and redundant degrees of freedom to perform a task. Recent studies have also suggested considering the task to compute inter-joint coordination metrics [88] as the task and its constraints directly affects the coordination strategy (i.e, different coordination strategies will be used if a glass must be placed upside down or right side up on a specific location).

**Angle extraction joint sequence** All the described metrics use joint angle trajectories that have been extracted from the different joints’ positions. In the tested dataset, joint angles correspond to the values recorded at the joint angles of the robotic exoskeleton, but according to the field in which the data will be used, they could have been extracted according to other specific joint sequences (International Society of Biomechanics (ISB) convention [85], external reference frame [89]...). The same movement but with angles extracted along different kinematic chain parametrization might yield different inter-joint coordination results. The result of the inter-joint coordination metric is dependent on the angle extraction kinematic chain, except for the Relative Joint Angle Correlation metric. With existing metrics, choosing carefully the kinematic chain from which angles will be extracted is a major point before measuring inter-joint coordination.

**Intrinsic rotation constraints** As detailed in the previous paragraph, extracting the angles is a critical step to then compute metrics. Generally, Euler angles are used. Euler’s angles are a sequence of 3 rotation angles used to describe the orientation of a rigid body in space with respect to a fixed coordinate system. However, Euler angles present various limitations. The first one is the extraction sequence. Euler’s angles are dependent on their rotation sequence, without this information, extracted angles are not comparable. Secondly, one unique orientation can be reached with different rotation sequences, this means that one rotation sequence needs to be chosen among the different possibilities, but there is no “right” choice. Depending on this choice, the metric’s result will vary. Finally, one last solution is to use quaternions. Quaternions are a mathematical tool that overcomes the different issues presented for Euler’s angles. However, from a physiological point of view quaternions are difficult to represent, and using quaternions add a level of complexity to the angle extraction process.

**Variability of the inter-joint coordination** The metrics listed here are trying to measure an inter-joint coordination pattern. Other metrics are measuring the variability of a relationship between joints [53, 90–92]. Combining measures of the inter-joint coordination with measures of the variability of the inter-joint coordination would open the results to more representative conclusions.

**Comparison with clinical data**

Comparing the results of metrics with clinical assessments (such as Fulg-Meyer Motor Assessment...) has already been done in other studies [24, 93]. In order to extend the results presented here and understand better how correlated or not metrics are with clinical tests, a similar study should be conducted.

**Metrics improvements** Almost all metrics underline interesting features of inter-joint coordination. However, some metrics could be improved or extended in order to facilitate analysis. For example, angle-angle plots can be extended using the angular coefficient of correspondence (slope of two consecutive points [64]).

Working on the visualization tools for the different metrics might also serve to make their findings more intelligible. Time delay metrics such as TC, ZC and ICI could be represented as histograms or as bar plots indicating mean and variance. When looking at 2 different conditions, the bar plot makes the comparison easier.

In order to make inter-joint coordination metrics more reliable, sensitive, and easier to interpret, combining several metrics in order to combine advantages of different metrics is another possibility. For example, PC analysis could be run on CRP data.

**Comparing metrics from different experiments** One main issue with coordination metrics is that from one experiment to the other, it’s almost impossible to compare the results obtained. By making the metrics more generic and dimensionless (using ratios and percentages, for example), it would be easier to compare the results between different experiments or tasks.

Creating meta-metrics could also be explored in order to be able to compare more easily inter-joint coordination. This would also facilitate comparison between metrics.

## Conclusion

Addressing the question of inter-joint coordination is a critical question in motion analysis. This notion is used in various fields and several definitions have been identified. Over the years, different metrics of inter-joint coordination for discrete movement tasks have been developed, each metric, highlighting one or several characteristics of inter-joint coordination. During this study, no singular metric set itself apart. Rather, depending on the phenomenon that needs to be observed, different metrics could be used. While no perfect metric for the analysis of inter-joint coordination exists, using several metrics might be a good approach to capture different characteristics of any given movement.

## Data Availability

Not applicable for a review.
